# Is preoperative emergency department utilization associated with postoperative healthcare utilization after total joint arthroplasty? A social vulnerability index based analysis

**DOI:** 10.1371/journal.pone.0343802

**Published:** 2026-02-25

**Authors:** John F. Burke, Corinne E. Vennitti, Wendy M. Novicoff, Matthew Oley, James A. Browne, Ian M. Duensing

**Affiliations:** University of Virginia Medical Center, Department of Orthopaedic Surgery, Charlottesville, Virginia, United States of America; Upperline Health Inc., UNITED STATES OF AMERICA

## Abstract

**Background:**

As the need for total joint replacement increases with an aging population, there is increased attention to perioperative healthcare utilization. Efforts to mitigate complexity which may lead to deviations from successful surgical outcomes are critical, now more than ever. Analysis of predictive variables associated with increased healthcare utilization postoperatively can aid the effort to decrease emergency department (ED) visits and overall burden to the healthcare system.

**Methods:**

A retrospective review was completed of all patients with total knee or total hip arthroplasty between 2017 and 2020 at a single institution. Inclusion criteria were patients who underwent primary, elective total knee or hip arthroplasty and received a minimum of one year follow up. Zip code was used to determine the social vulnerability index (SVI) for each patient. Preoperative ED visits were collected for 90 days and 12 months prior to surgery.

**Results:**

There were 1,059 patients included, 193 with an ED visit 12 months prior to surgery and 45 with an ED visit 90 days prior to surgery with an average SVI of 0.50. Analysis of the postoperative outcomes demonstrated 104 patients with ED visits within 90 days following the procedure. Comparisons between SVI and all preoperative and postoperative variables demonstrated no statistical significance. Preoperative ED visit within 90 days or 12 months of surgery was associated with increased likelihood of postoperative ED visit within 90 days (p < 0.001, OR 4.64; p < 0.001, OR 3.78). Preoperative ED visit within 90 days or 12 months was also associated with increased risk of readmission (p = 0.008, OR 6.09; p = 0.005, OR 3.80) and revision surgery or reoperation (p = 0.039, OR 3.57; p = 0.041, OR 2.20).

**Conclusion:**

Preoperative ED visit within 90 days or 12 months prior to surgery is a strong predictor of postoperative ED visit, readmission, and overall perioperative healthcare utilization regardless of socioeconomic factors.

## Introduction

There is a growing patient population in need of total joint replacement. Between 2000 and 2019, the annual volume of total hip arthroplasty (THA) increased 177% and total knee arthroplasty (TKA) increased by 156% on average [1]. Regression models have shown these rates will grow approximately 5% annually, with variability based on country, with models predicting up to 176% increase in THA and 139% increase in TKA by 2040 [1]. This increasing growth necessitates increased attention to healthcare utilization, access, and complications such as emergency department (ED) visits or readmission due to infection, revision surgery/reoperation, or postoperative medical complications.

Chief complaints frequently documented as reasons for return to the ED are deep venous thrombosis (DVT), postoperative pain control, wound concerns, anxiety, and/or other medical complications unrelated to surgical procedures [2–5]. Prior studies have demonstrated a 30-day ED visit rate of 5.2–11.0% and a 90-day ED visit rate of 5.0–19.1% for all total joint arthroplasty (TJA) patients [3,6,7]. Many institutions have developed avenues for patients to access providers and decrease ED utilization such as preoperative education packets, postoperative check-in telephone calls, online patient portals for communication with their surgeon, and after hour trainee/advanced practice provider on-call phone lines. However, despite attempts to mitigate these visits, there is a persistent and significant cost burden to the health care system with cost per ED visit after TJA averaging $429 and cost per readmission averaging $6,484 [8]. Additionally, a large number of these visits may be avoided or managed as an outpatient as 80–90% of these patients are discharged without readmission [7,9,10]. Prior studies have demonstrated the most common causes of ED visits in both 30-days and 90-days after TJA to be postoperative pain/swelling, wound concerns, and medication related side effects [3,7,8]. All other concerns such as trauma, cardiovascular, urological, pulmonary, social issues, and neurologic symptoms composed a small portion of the cohort analyzed [7].

Various studies assessing efficient healthcare utilization throughout the perioperative period have been conducted to identify areas of improvement or factors predictive of increased postoperative health system utilization. Previous literature has suggested predictive factors for increased healthcare utilization in the form of ED visits and readmission such as socioeconomic status, ethnicity, race, housing status, and medical comorbidities [11–15]. There is conflicting data to support a correlation between socioeconomic status and postoperative ED visits. Few studies have demonstrated higher social vulnerability index (SVI) scores in patients who returned to the ED within 90-days of discharge [12,13]. However, in a study by Shaw et.al, they found no correlation between area deprivation index (ADI) and likelihood of postoperative ED visits [11]. Other studies have demonstrated increased likelihood of postoperative ED visit if there was a preoperative ED visit within 12 months of TJA [16,17]. The purpose of this study was to determine if 1) a preoperative ED visit within 90 days or 12 months of TJA is associated with a postoperative ED visit, complications, or readmissions; 2) if SVI score as a measure of socioeconomic disadvantage is associated with a postoperative ED visit or complications; and 3) an orthopaedic specific concern as reason for preoperative ED visit is more or less likely to correlate with a postoperative ED visit or complications than non-orthopaedic complaints.

## Materials and methods

After institutional review board (IRB) exemption, a retrospective review was completed at a large, academic tertiary care center of all patients with TKA or THA between 2017 and 2020. Billing codes were used to identify patients prior to chart review. Data was accessed on 9/23/2022. Inclusion criteria were patients who underwent primary, elective total hip or knee arthroplasty and received a minimum of one year follow up. Patients who underwent revision joint replacement or underwent a total hip replacement secondary to femoral neck fracture were excluded. All procedures were performed by one of three fellowship trained adult reconstruction surgeons.

Demographic variables at the time of operation were obtained via chart review, including age, sex, date of surgery, date of death (if applicable), and zip code. Patient zip code at time of surgery was used to calculate the social vulnerability index using the SVI interactive map compiled by the geospatial research analysis and services government program through the Center for Disease Control [18]. The map was originally designed to identify communities that may require additional support in times of disasters. This map was created using census variables from the 5-year American Community Survey to establish a grading system based on socioeconomic status, household characteristics, racial and ethnic minority status, and housing type and transportation. The assessment of these four categories, in a particular zip code, are combined to provide a single measurement of social vulnerability for a particular geographic area. Higher scores on this scale represent greater social vulnerability. This data is collected using comprehensive, yearly census data and provides SVI based on year, allowing a more accurate representation of the environment of the patient population at the time of surgery. SVI was chosen for this study as it provides a multifactorial assessment of social vulnerability and has been utilized previously in orthopaedic and medical literature.

Preoperative variables included ED visits within one year prior to total joint arthroplasty. Each ED visit was noted to have an orthopaedic related complaint or non-orthopaedic complaint. Of the orthopaedic complaints, visits that were associated with the operative extremity were noted. Postoperative variables including dates of postoperative clinic visits up to one year, postoperative ED visits within 90 days of operation, cause of postoperative ED visit, readmission within 90 days of operation, prosthetic joint infection (PJI) within 1 year, revision surgery or reoperation at last follow up, and overall mortality at last follow up were recorded.

There were 1,059 patients in the cohort for analysis. There were 480 patients that underwent THA and 579 patients that underwent TKA. The average age of the cohort was 65 years old with 59% of patients being women and 41% being men (Table 1). The average SVI of 0.50 indicated a medium level of vulnerability. There were 193 (18.2%) patients with an ED visit within 12 months prior to their procedure with the average number of ED visits prior to the procedure being 1.63. Of the 193 patients, 57 (29.5%) had a preoperative ED visit for orthopaedic related complaints with 36 (18.7%) of those related to their operative joint. There were 45 (4.2%) patients with an ED visit within 90 days prior to the procedure. Of these visits, 7/45 (15.5%) were for an orthopaedic complaint and 4/45 (8.9%) were related to their operative joint (Table 2).

**Table 1:** Demographic characteristics of the cohort including both total knee and total hip arthroplasty (n = 1059).

Variable	Cohort Analysis
Gender	
*Women*	628/1059 (59.3%)
*Men*	431/1059 (40.7%)
Age (years)	65 ± 10.7
ED visit within 12 months prior to procedure	193/1059 (18.2%)
*ED visit within 90 days prior to procedure*	45/193 (4.2%)
Number of ED visits prior to procedure*	1.63 ± 1.32
Procedure type	
*Total hip arthroplasty*	480/1059 (45.3%)
*Total knee arthroplasty*	579/1059 (54.7%)
Social Vulnerability Index (SVI)	0.50 ± 0.26

*This information was derived only from patients that had an ED visit prior to procedure (N = 193).

**Table 2:** Demonstration of pre-operative ED visits within 90 days and 12 months prior to surgical intervention and within 90 days post procedure with subgroup analysis by chief complaint.

Type of ED Visit	Number of Patients
ED visit 90 days pre-op	
Non-orthopaedic complaint	38/45 (84.4%)
Orthopaedic complaint	7/45 (15.6%)
ED visit 12 months pre-op	
Non-orthopaedic complaint	136/193 (70.5%)
Orthopaedic complaint	57/193 (29.5%)
ED visit 90 days post-op	
Non-orthopaedic complaint	53/104 (51%)
Orthopaedic complaint	51/104 (49%)

### Data analyses

Comparisons between SVI and all preoperative and postoperative variables were conducted using two sample t-tests; distributions were tested for normality using the Shapiro-Wilk test to confirm that parametric testing could be used. Comparisons between patients with a preoperative ED visit at 90 days and postoperative outcomes including postoperative ED visit were completed using Fisher’s Exact Tests. Comparisons between patients with a preoperative ED visit within 12 months and postoperative outcomes including postoperative ED visit were also completed using Fisher’s Exact Tests. All p-values less than 0.05 were considered statistically significant.

## Results

Analysis of the postoperative outcomes demonstrated 104 (9.8%) patients with ED visits within 90 days following their operation. Of these 104 patients, 51 (49%) had an orthopaedic concern, of which 45 (43.3%) was a concern related to their operative joint and 53 (51%) had non-orthopaedic concerns as cause for their ED visit. Of note, 31/104 (29.8%) of these patients had an ED visit due to pain related to their procedure. Other postoperative complications included 31 (2.9%) patients who underwent revision surgery or reoperation by time of last follow-up, 8 (0.8%) patients with PJI at one year, 20 (1.9%) patients with postoperative readmission to an inpatient unit within 90 days, and 38 (3.5%) mortalities at time of last follow up. There was no statistically significant relationship between SVI and postoperative ED visits, readmission, PJI, revision surgery/reoperation, or mortality (Table 3).

**Table 3:** Relationship between SVI score and postoperative outcomes (two-sample t-tests; n = 1059).

Outcome	Mean (SD) SVI for patients with outcome	Mean (SD) SVI for patients without outcome	p-value	Cohen’s d
PJI at 12 months	0.486 (0.27)	0.501 (0.26)	0.387	0.057
Reoperation/Revision	0.508 (0.26)	0.501 (0.26)	0.864	0.027
Post-operative ED visit within 90 days	0.522 (0.28)	0.501 (0.26)	0.412	0.078
Readmission within 90 days	0.460 (0.30)	0.503 (0.27)	0.549	0.151
Mortality	0.561 (0.25)	0.499 (0.26)	0.178	0.241

There were 45 patients who met the criteria of ED visit within 90 days of the procedure. There was a statistically significant association between ED visit within 90 days prior to the procedure and a postoperative ED visit within 90 days (p < 0.001), readmission within 90 days (p = 0.008), and revision/reoperation (p = 0.039) (Table 4). There was no statistically significant relationship between preoperative ED visit within 90 days and PJI (p = 0.294) or mortality (p = 0.073).

**Table 4:** Results of Fisher’s Exact Tests comparing outcomes of patients with a preoperative ED visit within 90 days of procedure (n = 45 patients) to patients who did not have a preoperative ED visit within 90 days of procedure (n = 1014).

Outcome	ED visit within 90 days preop	No ED visit within 90 days preop	p-value	OR (95% confidence interval)
PJI at 12 months	1/45 (2.2%)	7/1014 (0.7%)	0.294	3.27 (0.39-27.16)
Reoperation/Revision	4/45 (8.9%)	27/1014 (2.7%)	**0.039**	**3.57 (1.19-10.67)**
Post-operative ED visit within 90 days	14/45 (31.1%)	90/1014 (8.9%)	**<0.001**	**4.64 (2.38-9.04)**
Readmission within 90 days	4/45 (8.9%)	16/1014 (1.6%)	**0.008**	**6.09 (1.95-19.01)**
Mortality	4/45 (8.9%)	34/1014 (3.4%)	0.073	2.81 (0.95-8.30)

There were 193 patients who met the criteria of ED visit within 12 months of the procedure. There was a statistically significant association between an ED visit within 12 months prior to procedure and postoperative ED visit within 90 days (p < 0.001), readmission (p = 0.005), revision/reoperation (p = 0.041), and mortality (p = 0.031) (Table 5). There was no statistically significant association between ED visit within 12 months prior to procedure and PJI (p = 0.643). Subgroup analysis was performed for patients presenting to the ED within 12 months prior to procedure due to orthopaedic concerns (n = 57). There was no statistically significant relationship between presentation to the ED for an orthopaedic concern and postoperative ED visits, readmission, revision/reoperation, PJI, or mortality (Table 6). Table 7 contains the results for cross-tabulation of frequency of preoperative ED visits within 12 months separated into groups (i.e., 1, 2, or ≥3 preoperative visits) and postoperative ED visit within 90 days, which demonstrates a statistically significant association between increasing number of preoperative visits and postoperative ED visit (p < 0.001).

**Table 5:** Results of Fisher’s Exact Tests comparing outcomes of patients with a preoperative ED visit within 12 months of procedure (n = 193 patients) to patients who did not have a preoperative ED visit within 12 months of procedure (n = 866).

Outcome	ED visit within 12 months	No ED visit within 12 months	p-value	OR (95% confidence interval)
PJI at 12 months	2/193 (1.0%)	6/866 (0.7%)	0.643	1.50 (0.30-7.49)
Reoperation/Revision	10/193 (5.2%)	21/866 (2.4%)	**0.041**	**2.20 (1.02-4.75)**
Post-operative ED visit within 90 days	43/193 (22.3%)	61/866 (7.0%)	**<0.001**	**3.78 (2.47-5.80)**
Readmission within 90 days	9/193 (4.7%)	11/866 (1.3%)	**0.005**	**3.80 (1.55-9.31)**
Mortality	12/193 (6.2%)	26/866 (3.0%)	**0.031**	**2.14 (1.06-4.33)**

**Table 6:** Results of Fisher’s Exact Tests comparing outcomes of patients with a preoperative ED visit related to an ortho complaint within 12 months of procedure (n = 57 patients) to patients who did not have a preoperative ED visit related to an ortho complaint within 12 months of procedure (n = 1002).

Outcome	ED visit for orthopaedic complaints within 12 months preop	No ED visit for orthopaedic complaints within 12 months preop	p-value	OR (95% confidence interval)
PJI at 12 months	2/57 (2.2%)	6/1002 (0.6%)	0.641	3.25 (0.41-25.42)
Reoperation/Revision	4/57 (7.0%)	27/1002 (2.7%)	0.080	2.73 (0.92-8.07)
Post-operative ED visit within 90 days	10/57 (17.5%)	94/1002 (9.4%)	0.062	2.06 (1.00-4.20)
Readmission within 90 days	1/57 (1.8%)	19/1002 (1.9%)	0.707	0.924 (0.12-7.03)
Mortality	4/57 (7.0%)	34/1002 (3.4%)	0.143	2.15 (0.74-6.28)

**Table 7:** Cross-tabulation of increasing frequency of preoperative ED visits within 12 months of procedure and postoperative ED visit within 90 days. Chi-square analysis p-value <0.001.

	No ED visit postop	ED visit postop	Total
0 ED visits preop	805	61	866
1 ED visit preop	111	24	135
2 ED visits preop	22	8	30
≥3 ED visits preop	17	11	28
Total	955	104	1059

## Discussion

Despite improvements in access to care, there still exists a significant portion of patients who present to the ED for evaluation postoperatively. The importance of decreased hospital care episodes following TJA has been highlighted with the establishment of the Comprehensive Care for Joint Replacement Model set in place in 2016 [19,20]. This model offers incentives for hospitals and providers to encourage coordinated, comprehensive care while carrying penalties for increased healthcare utilization in the postoperative period [19,20]. Within this model, one global payment is used to cover the entire perioperative period, further emphasizing the goal to decrease unnecessary postoperative costs [19–21]. In the present study of 1,059 patients undergoing total joint arthroplasty, 104 patients visited the ED within 90 days postoperatively yielding an incidence of 9.8%, which is similar to the prior reported incidence of a 90-day ED visit rate of 5.0–19.1% for all TJA patients [3,6,7]. In the analysis of our patient population, preoperative ED visit within 90 days or 12 months was strongly predictive of postoperative ED visit within 90 days. Interestingly, this study demonstrated that preoperative ED visit within 90 days or 12 months prior to surgery was a risk factor for readmission within 90 days and revision surgery/reoperation, which has not been reported in prior studies. There was no association found between SVI and postoperative ED visits, readmissions or other complications.

Prior studies have attempted to determine risk factors for postoperative ED visits after TJA. A large database study demonstrated increased risk of postoperative ED visit following TKA in older patients, men, lower income, increasing comorbidities, longer inpatient length of stay, and not having a post op visit with a primary care provider [22]. Another large database study identified increasing age and higher Elixhauser Comorbidity Index scores as risk factors for ED visits following TKA [4]. Additionally, preoperative ED visits within 12 months prior to surgery have been shown to be associated with increased risk of postoperative ED visits following TKA or THA, with an odds ratio of 2.0 in one study [11,16,17]. The present study supports these findings, and also showed that increasing frequency of preoperative ED visits (i.e., 1, 2, or ≥3) was associated with an increased likelihood of postoperative ED visit. The subgroup analysis of patients with a preoperative ED visit for an orthopaedic concern was not predictive of postoperative complications or post op ED visits.

There have been many hypotheses regarding socioeconomic, racial/ethnic, and medical variables impacting postoperative ED utilization. Prior literature has noted that race, housing status, access to healthcare, access to primary care providers, depression, drug abuse, insurance provider, and psychiatric conditions have all been found to correlate with postoperative ED visits [5,13–15,23,24]. However, there is heterogeneity in the literature regarding composite measures for socioeconomic status and social vulnerability and correlation to postoperative outcomes. In a study by Baxter et. al, a higher overall SVI score and the household characteristics dimension of the SVI score were independent risk factors for postoperative ED visit within 90 days following TKA but not THA [12]. They did not find any association between SVI and readmission or other postoperative complications [12]. Shaw et al. investigated if there was any predictive power of area deprivation index (ADI), which is a composite measure used to quantify socioeconomic risk based on location, and early postoperative ED visits and found no statistically significant risk utilizing this measure [11]. Additionally, Diaz et al. found that SVI was not associated with any postoperative complication, mortality, or readmission after lower extremity joint replacement [25]. This differed from what they found regarding an association between SVI and negative postoperative outcomes following colectomy, lung resection, or coronary artery bypass graft [25]. Furthermore, a prior large registry study found higher SVI and ADI quartiles to be associated with increased rate of discharge to a skilled nursing facility, increased postoperative ED visits and readmissions, and aseptic revision [13]. Based on the scores collected for our patient population, there was no statistically significant association between SVI and likelihood of postoperative ED visit, readmission, or other postoperative variables.

There are several limitations to this study. The use of SVI as a representation of socioeconomic status has variable results for accuracy in the literature and should be assessed with caution. SVI is composed of both modifiable and nonmodifiable components, and there are many factors that are not included in SVI such as transportation, crime rates, and health literacy [26]. The literature has demonstrated that not all components of the SVI correlated positively with each assessed complication after TJA and each should be considered separately [26,27]. Another limitation is the lack of control for potential confounding variables including medical comorbidities. Patients presenting to the ED preoperatively more frequently for non-orthopaedic concerns may have more medical comorbidities that would predispose them to postoperative ED visits and postoperative complications. Additionally, patients with more frequent preoperative ED visits may lack access to a primary care physician, and therefore, may be more likely to have more postoperative visits as well. The subgroup analysis including the orthopaedic versus non-orthopaedic related reasons for preoperative ED visit and the measured outcomes was likely underpowered for accurate analysis. Length of follow up is another limitation of this study, as the data collected required a minimum of 12 month follow up, which may inaccurately represent overall revision/reoperation and PJI rates.

## Conclusion

With the increasing number of total joint arthroplasties performed and rising cost of healthcare, the identification of factors impacting postoperative healthcare utilization is needed to create pathways to mitigate unnecessary burden on the healthcare system. The presence of a preoperative visit, orthopaedic related or not, within 90 days or 12 months prior to surgery is a strong predictor of postoperative ED visits and readmission within 90 days, regardless of socioeconomic factors, as shown by the lack of correlation between SVI and postoperative ED visits. This association can be used to aid in identification of patients who may require more targeted pre-operative teaching, closer in-person or virtual follow-up, and potentially delaying or optimizing these patients in attempts to avoid unnecessary ED utilization postoperatively.
